# Case report: Secondary neck mass infection in a patient with multiple osteomyelitis caused by *Corynebacterium striatum*

**DOI:** 10.1097/MD.0000000000043902

**Published:** 2025-08-29

**Authors:** Xiang Chen, Jian-Hui Zhang, Jing-Yi Chen, Shu-Guang Chen, Jie-Wei Luo, Sheng-Xun Mao

**Affiliations:** a The First Clinical Medical College, Nanchang University, Nanchang, China; b Department of Traditional Chinese Medicine, Shengli Clinical Medical College of Fujian Medical University, Fujian Provincial Hospital, Fuzhou, China; c Department of Emergency Surgery, Fuzhou University Affiliated Provincial Hospital, Fuzhou, China; d Department of Gastrointestinal Surgery, The Second Affiliated Hospital of Nanchang University, Nanchang, China.

**Keywords:** case report, *Corynebacterium striatum*, multiple osteomyelitis, secondary infection

## Abstract

**Rationale:**

The opportunistic pathogen *Corynebacterium striatum* has been generating more clinical infections in recent years, but secondary infections at different parts caused by it have been reported more rarely.

**Patient concerns:**

This case details a 52-year-old male patient who got an infection that advanced to multiple osteomyelitis and soft tissue abscess after block therapy for external humeral epicondylitis of the right arm. Unexpectedly, the chronic ulcerated region of the patient’s neck was infested with *Corynebacterium striatum* due to inadequate treatment of the main infection, resulting in a secondary infection of the neck mass.

**Diagnoses:**

Microbiological cultures of the pus from the right elbow and neck indicated an infection with *Corynebacterium striatum* at both sites.

**Interventions:**

We initiated antimicrobial therapy with linezolid (600 mg) in conjunction with Fosfomycin (4 g) every 12 hours.

**Outcomes:**

After 4 weeks of treatment, the infected lesion was resolved as evidenced by a repeat magnetic resonance imaging compared to the prior scan.

**Lessons:**

This case not only demonstrates the significant pathogenicity of *Corynebacterium striatum* but also warns that traumatic locations, such as swellings resulting from chronic ulcers in the neck, may become prospective targets for this bacterium.

## 1. Introduction

*Corynebacterium striatum*, previously regarded as a contaminant, has recently been recognized as an infectious agent due to a rise in reported infections, particularly among immunocompromised individuals.^[1]^ Infection with *C striatum* may manifest as septicopyemia, endocarditis, and osteoarthritis.^[2–4]^ Although *C striatum* infections exhibit varied clinical symptoms, cases of secondary infections are rare. In clinical practice, *C striatum* is frequently perceived as a contaminant rather than a pathogenic agent, leading to several infections being ignored or misdiagnosed, hence delaying appropriate treatment. This case presents a 52-year-old male patient with multiple osteomyelitis and a subsequent infection of a neck mass attributed to *C striatum* following block therapy for external humeral epicondylitis. This investigation examines the possible origins of secondary infections attributed to *C striatum*, cautioning that traumatized sites, such as a mass resulting from a chronic neck ulcer, may become susceptible to the bacterium. It emphasizes the necessity for more vigorous treatment of other wounds or uninfected lesions in the patient.

## 2. Case report

**Figure 1.**
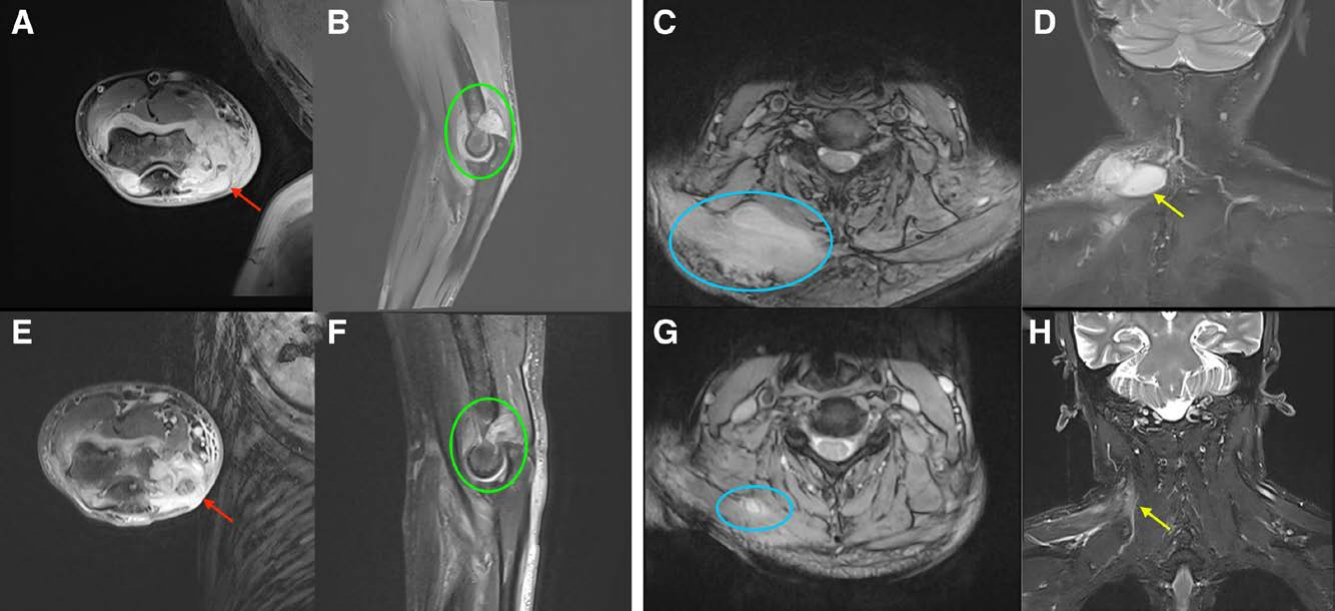
Patient MRI results. (A and B) Inflammatory edema of the right elbow’s distal, radial, and ulnar humeri, possibly due to osteomyelitis; inflammation and exudation of the soft tissues around the elbow joint, with abscess formation; multiple fluid and pus accumulations in the elbow joint cavity and bursa; (C and D) infectious lesions were suspected in the subcutaneous area of the right back of the neck with patchy T2WI high signals; (E and F) abscesses on the right elbow were reduced following therapy; (G and H) neck post-treatment results showed considerable improvement in infectious lesions. (Red arrows and green circles show right elbow alterations before and after infection treatment; yellow arrows and blue circles show neck changes) MRI = magnetic resonance imaging.

**Figure 2.**
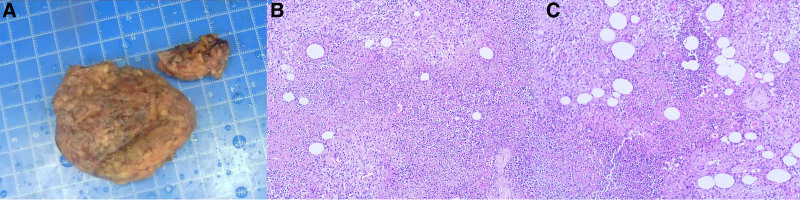
(A–C) The pathological examination of the patient’s neck mass revealed pyogenic granulomatous alterations.

The patient is a 52-year-old male who received localized block therapy for external humeral epicondylitis in the right arm and subsequently experienced severe swelling, localized elevated skin temperature, and fever at the therapy site 2 months later. Notably, the patient has a chronic ulcer on his neck, a residual effect of an acupuncture treatment from 1 year ago. Routine blood tests revealed significantly higher leukocyte and neutrophil counts, along with increased levels of the inflammatory markers C-reactive protein and procalcitonin; however, microbiological cultures of both blood and pus yielded negative results. The magnetic resonance imaging (MRI) revealed inflammation and edema of the distal humerus, radial head, and ulnar humerus of the right elbow, indicative of osteomyelitis; inflammation and exudation of the soft tissues surrounding the right elbow joint, accompanied by partial abscess formation; and multiple effusions and pus accumulations within the joint cavity of the right elbow and the adjacent bursa (Fig. 1A and B). We promptly executed incision and drainage of the right elbow abscess combined with a vacuum-sealing drainage, and empirically supplied cefoperazone sulbactam (2 g) alongside ornidazole (0.5 g) every 12 hours to combat infection. Postoperatively, the patient exhibited ongoing chills and fever. Four weeks postsurgery, swelling in the ulcerated region of the neck was noted, characterized by firmness upon palpation and significant tenderness. An MRI of the neck indicated a patchy T2-weighted imaging high signal in the right posterior cervical subcutaneous area, suggestive of infection (Fig. 1C and D). Microbiological cultures of the pus from the right elbow and neck indicated an infection with *C striatum* at both sites. We subsequently performed surgery on the neck mass, measuring approximately 6 × 5 × 4 cm, characterized by chronic ulcerated hyperplastic tissue containing yellowish pus. This tissue was attached to the posterior neck muscles and ligaments, and postoperative pathology revealed pyogenic granulomatous alterations (Fig. 2A–C). We initiated antimicrobial therapy with linezolid (600 mg) in conjunction with Fosfomycin (4 g) every 12 hours. After 4 weeks of treatment, the patient exhibited no further fever, blood parameters and inflammatory markers normalized, and the infected lesion was resolved as evidenced by a repeat MRI compared to the prior scan (Fig. 1E–H). The patient discontinued antibiotics and had anti-inflammatory treatment with Chinese medicines in the outpatient clinic, which proved beneficial and resulted in no recurrence.

## 3. Discussion

*C striatum* is a non-spore-forming, aerobic, gram-positive bacterium belonging to the *Corynebacterium* family, commonly found on human skin and in the respiratory tract. Recent research has recognized it as a significant conditional pathogen capable of inducing clinical illnesses.^[5]^ *C striatum* infection is typically associated with susceptibility factors, including age and preexisting conditions.^[1]^ Moreover, invasive diagnostic procedures and treatments, extended administration of broad-spectrum antibiotics, and prolonged hospitalization have been recognized as significant risk factors for *C striatum* infection.^[2,6]^ This case describes a patient who received intrusive block therapy, leading to a localized infection of *C striatum* in the right elbow, resulting in multifocal osteomyelitis and peripheral soft tissue suppuration. We contend that the presence of *C striatum* in the microbiological culture of the neck mass is not coincidental, suggesting 2 potential explanations: External skin invasion: Chronic ulcers compromise the integrity of the skin or mucous membranes, creating an entry point for pathogens such as *C striatum*, a conditional pathogen. In patients with extended hospital stays and prolonged use of ineffective antibiotics, *C striatum* can exploit the microenvironment of the chronic ulcer to proliferate rapidly and induce local infection; the right elbow as the primary lesion disseminates via the internal circulation: In instances where initial empirical antimicrobial treatment fails to control the infection, postoperative chills and fever may result from pathogenic organisms entering the bloodstream and migrating to the neck mass. Furthermore, chronic ulcers frequently coincide with persistent inflammation and immunological reactions in localized tissues, and the extended inflammatory response may result in localized immune cell depletion and diminished immune function. This facilitates bacterial colonization of the ulcer site and enables evasion of the host’s immune clearance processes.

The culture results for *C striatum* are clinically contentious, and when positive, the organism is typically regarded as a contaminant rather than the etiological agent, leading to the oversight of numerous *C striatum* infections in clinical settings.^[1,7]^ The recurrent isolation of *Corynebacterium spp. strains* from sputum and blood specimens must be treated with due seriousness and analyzed in conjunction with clinical findings rather than dismissed as contaminants. The potential pathogenicity of *C striatum* should be acknowledged to prevent delays in diagnosis and treatment.^[8]^ This case indicates the significance of conducting repeated cultures of wound secretions, prompt management, and the judicious selection of reasonable antibiotics to address the main infection of multiple osteomyelitis to diminish bacterial load and mitigate transmission risk. Secondly, it is crucial to recognize that patients infected with *C striatum* who have other wounds require vigorous local treatment; otherwise, they may become susceptible to invasion and colonization by *C striatum*.

## Author contributions

**Conceptualization:** Sheng-Xun Mao.

**Data curation:** Xiang Chen.

**Formal analysis:** Jing-Yi Chen.

**Supervision:** Shu-Guang Chen, Sheng-Xun Mao.

**Writing – original draft:** Jian-Hui Zhang, Xiang Chen.

**Writing – review & editing:** Jie-Wei Luo.

## References

[1] Silva-Santana G Silva CMF Olivella JGB. Worldwide survey of Corynebacterium striatum increasingly associated with human invasive infections, nosocomial outbreak, and antimicrobial multidrug-resistance, 1976-2020. Arch Microbiol. 2021;203:1863–80. 33625540 10.1007/s00203-021-02246-1 PMC7903872

[2] Yamamuro R Hosokawa N Otsuka Y Osawa R. Clinical characteristics of corynebacterium bacteremia caused by different species, Japan, 2014-2020. Emerg Infect Dis. 2021;27:2981–7. 34812137 10.3201/eid2712.210473 PMC8632174

[3] Doub JB. Is Corynebacterium striatum an emerging prosthetic joint infection pathogen and how should it be treated? Germs. 2023;13:151–7. 38144248 10.18683/germs.2023.1378 PMC10746339

[4] Usuda D Kojima Y Ono R. Native valve endocarditis caused by Corynebacterium striatum without underlying structural heart disease or indwelling cardiovascular medical devices: a case report. BMC Infect Dis. 2024;24:939. 39251918 10.1186/s12879-024-09825-9 PMC11384686

[5] Mangutov EO Kharseeva GG Alutina EL. Corynebacterium spp. – problematic pathogens of the human respiratory tract (review of literature). Klin Lab Diagn. 2021;66:502–8. 34388322 10.51620/0869-2084-2021-66-8-502-508

[6] Milosavljevic MN Milosavljevic JZ Kocovic AG. Antimicrobial treatment of Corynebacterium striatum invasive infections: a systematic review. Rev Inst Med Trop Sao Paulo. 2021;63:e49. 34161555 10.1590/S1678-9946202163049 PMC8216692

[7] Von Graevenitz A Punter-Streit V Riegel P Funke G. Coryneform bacteria in throat cultures of healthy individuals. J Clin Microbiol. 1998;36:2087–8. 9650969 10.1128/jcm.36.7.2087-2088.1998 PMC104985

[8] Ishiwada N Watanabe M Murata S Takeuchi N Taniguchi T Igari H. Clinical and bacteriological analyses of bacteremia due to Corynebacterium striatum. J Infect Chemother. 2016;22:790–3. 27654073 10.1016/j.jiac.2016.08.009

